# Efficacy of artemether-lumefantrine for the treatment of uncomplicated *Plasmodium falciparum* malaria in Nepal

**DOI:** 10.1186/s40794-018-0068-2

**Published:** 2018-08-14

**Authors:** Prakash Ghimire, Komal Raj Rijal, Chandramani Kafle, Balman Singh Karki, Nihal Singh, Leonard Ortega, Garib Das Thakur, Bipin Adhikari

**Affiliations:** 1 Central Department of Microbiology, Tribhuvan University, Kirtipur, Kathmandu, Nepal; 2 KIST Medical College, Gawrko, Lalitpur, Nepal; 3 World Health Organization, Country office Nepal, UN House, Pulchowk, Lalitpur, Nepal; 4 Global Malaria Program, World Health Organization headquarters, Geneva, Switzerland; 5 Ministry of Health, Government of Nepal, Kathmandu, Nepal; 6 Mahidol Oxford Tropical Medicine Research Unit, Faculty of Tropical Medicine, Mahidol University, Bangkok, Thailand

**Keywords:** Artemether-lumefantrine, ACT, Treatment, Uncomplicated falciparum, Malaria, Nepal

## Abstract

**Background:**

The national treatment guidelines of Nepal have adopted Artemisinin Combination Therapies (ACTs) for the treatment of uncomplicated falciparum malaria since 2004. Emergence of Artemisinin resistance in the Greater Mekong Sub-region (GMS) and beyond may become a threat for Nepal as well. The main objective of this study was to assess the therapeutic efficacy of antimalarial drug artemether-lumefantrine in uncomplicated *P. falciparum* infected patients at health centers/hospitals treated over the period of 2 years (2013–2014).

**Methods:**

Giemsa stained thick and thin smears, prepared from uncomplicated falciparum malaria patients who visited the selected sentinel sites in Nepal during 2013 to 2014 and met the inclusion criteria that included parasitemia (1000–10,000 /μL of blood), were evaluated until 28 days after ACTs treatment, following a World Health Organization (WHO) therapeutic efficacy protocol. Based on the re-occurrence of fever and resurge in parasitemia, the study patients were classified as resistant or susceptible. Blood specimens on filter papers were further analyzed by Polymerase Chain Reaction (PCR), specifically for the K13 propeller gene mutation (a recently identified molecular marker for ACT resistance).

**Results:**

A total of 56,013 suspected malaria cases were screened for this study. Of which, 120 (0.21%) were infected with falciparum malaria. Out of 120, 28 cases of *P. falciparum (*28/120; 23.33%) were enrolled in the study, of which 24 cases completed the post-treatment follow up for 28 days. Only one case out of 24 (4%) was identified as a late treatment failure (LTF). K13 mutation, a proxy indicator for ACT resistance in parasites, was not detected on the day 1, which indicates resistance had not yet reached the molecular level.

**Conclusion:**

Only one case of late treatment failure was identified in this study. ACT combination using artemether-lumefantrine was still effective for the treatment of uncomplicated falciparum malaria in Nepal. A close monitoring and supervision for ACT resistance is essential for future malaria treatment in Nepal.

## Background

Malaria is a global public health problem caused by *Plasmodium* species [1]. According to the World Health organization (WHO), in 2016 it was estimated that 445,000 people died from malaria and 216 million people were infected with malaria. [2]. However, the number of deaths related to malaria is declining significantly each year [2]. Among the five types of malaria [3], falciparum malaria, caused by *P. falciparum*, is the deadliest one [4] on the basis of its severity and complications.

Artemisinin or its derivatives or Artemisinin in combination with other drugs, is used as a first line drug for the treatment of uncomplicated falciparum malaria in many countries [5]. In Nepal, a combination of artemether and lumefantrine—called as Artemisinin Combination Therapies (ACTs) have been used as a first line drug for the treatment of falciparum malaria since 2004 [6, 7]. ACTs are effective [8] and there have been no remarkable severe adverse events reported to date. However, the emergence of ACTs resistance in GMS and its potential spread may become a public health disaster [9, 10]. Malaria elimination efforts are currently underway in the GMS to contain its spread westward [11–17]. However, continuous monitoring of efficacy of antimalarials (ACTs) is critical as malaria control programs in nations including Nepal rely on it as a first line treatment.

Assessment of therapeutic efficacy entails in-vivo measurements of parasitemia in blood, coupled with the monitoring of clinical symptoms in patients undergoing the treatment with ACTs for 28 days [18]. This remains the standardized test for the assessment of drug resistance in *Plasmodium falciparum*. Clinical symptoms monitoring is classified as early treatment failure (ETF), late treatment failure (LTF), late parasitological failure (LPF) and adequate clinical and parasitological response (ACPR).

ETF is defined as the presence of danger signs or severe malaria on day 1, 2 or 3 including parasitemia. LTF constitutes late clinical failure and late parasitological failure. Late clinical failure is defined as presence of danger signs or severe malaria including parasitemia on any day between day 4 and day 28. Late parasitological failure is defined as the presence of parasitemia on any day between day 7 and 28. ACPR is defined as absence of parasitemia by the end of the treatment (day 28) irrespective of axillary temperature without previously meeting any of the criteria of early treatment failure or late clinical failure or late parasitological failure [19–21]. Anti-malarial drug resistance in uncomplicated falciparum is characterized by either ETF or LTF [22].

Late treatment failure by microscopy needs additional monitoring for Artemisinin resistance by PCR. Artemisinin resistance is specifically associated with mutation on Klech 13 (K13) propeller region of the parasite and therefore, identification of this is essential [23]. Range of mutations, predominantly C580Y, on the propeller region of Kelch 13 protein was found to be responsible for slow parasite clearance time and thus resistance against Artemisinin combination drug [10, 24, 25].

In the current context of emergence of Artemisinin resistance around the region, and the burden of malaria being mostly (45–60%) imported in Nepal from migrant workers in south and south east Asia, it is critical to assess and monitor the efficacy of ACTs. There have not been any studies in Nepal to explore the efficacy and resistance of ACTs. The main objective of this study was to assess the efficacy and resistance pattern of ACTs currently recommended by national malaria control program.

## Methods

**Fig. 1:**
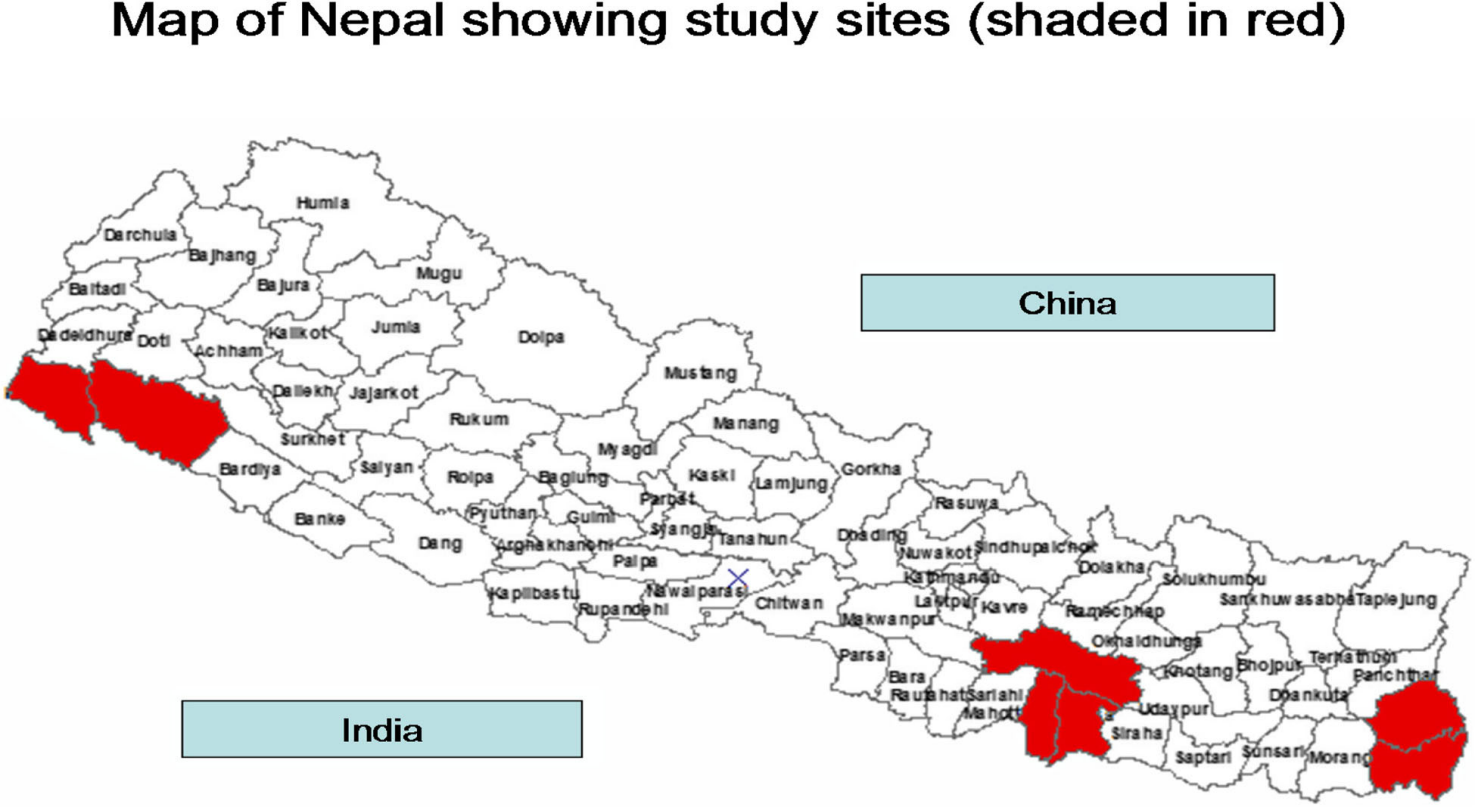
Study Sites

This study was conducted in four Zonal Hospitals (*Seti* and *Mahakali*- far western region, *Janakpur*- central region and *Mechi*- eastern region) with the catchment of patients from various districts (*Kailali, Kanchanpur, Dadeldhura, Doti, Bajura, Achham, Baitadi* and *Darchula*) (Fig. 1). The study was carried out between April 2013 and December 2014.

This study was an open-arm prospective exploration of clinical and parasitological responses based on the WHO therapeutic efficacy protocol-2009 and past research [21, 26]. All patients with uncomplicated malaria treated under ACTs (artemether-lumefantrine) were enrolled into the study. Patients with uncomplicated falciparum malaria who met the study inclusion criteria (axillary temperature ≥ 37.5 °C, aged above 6 months, parasitemia of 1000–10,000 /μL of blood and provided consent to participate) were screened and enrolled for study. Study enrollment began with hospitalization of the participants for the first 3 days (treated with artemether-lumefantrine regimen) and were followed on the 7th day and every week for 28th days. Patients with severe falciparum malaria, mixed infections, severe malnutrition, severe diseases, and pregnancy were excluded from the study. Additional follow ups were made if patients developed any relevant signs and symptoms until the end of the study period.

All patients received standard treatment at the hospital. Treatment with artemether-lumefantrine (each Coartem tablet containing artemether 20 mg and lumefantrine 120 mg) was provided based on the national malaria treatment protocol. Coartem was provided for a total of 3 days based on the body weight of the patients (5-14 kg: 1 tablet/day, 15-24 kg: 2 tablets/day, 25-34 kg: 3 tablets/day and > 35 kg: 4 tablets/day).

Clinical data included a standard physical examination report that included body weight and axillary temperature. Thick and thin blood films for parasite counts were obtained and screened on day 0 to confirm adherence to the inclusion criteria. Parasite counts were examined using thick smear on day 0, 1, 2, 3, 7, 14, 21 and 28 days (follow up period). Adverse events such as severe anemia, black water urine, hypoglycemia, abnormal bleeding, acidosis, and hemoglobinuria were monitored throughout the follow up period.

Molecular analysis for K13 mutation, using PCR, was recorded and further analyzed to confirm the presence of this mutation in parasites. Dried blood samples were collected onto Whatman filter paper No.1 and stored at room temperature in zip-lock plastic bags containing silica gel desiccant beads for further molecular analysis. The collected filter paper spots were sent to Mahidol University through the World Health Organization South East Asia Regional Office (WHO SEARO), for molecular analysis of the resistance targeting towards K13 mutation, as an indicator of resistance development.

## Result

**Fig. 2:**
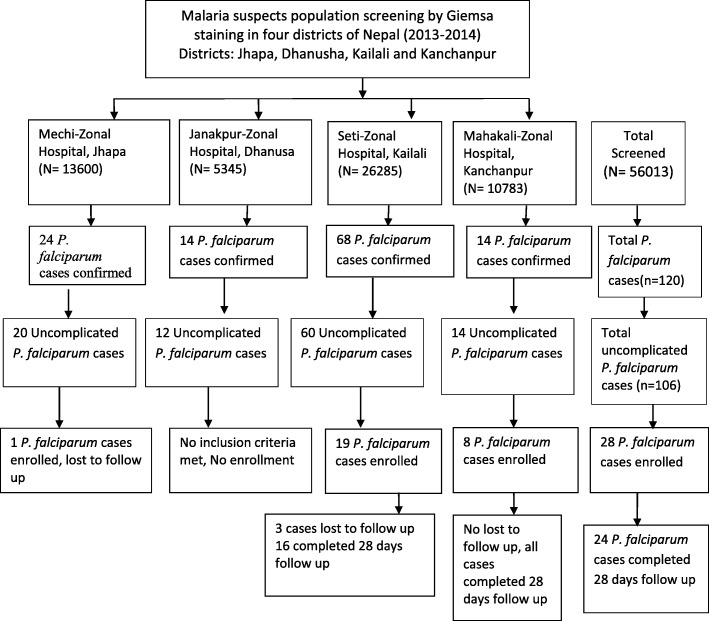
Trial flow of the study

A total of 56,013 febrile patients suspected for malaria were screened for the study. A total of 1447 (2.5%) were confirmed as malaria. Out of 1, 447, one hundred and twenty cases were confirmed with *P. falciparum.* Among 120 cases, 14 were excluded because of severe malaria (Fig. 2). One hundred and six patients were infected with uncomplicated falciparum malaria confirmed by microscopy and existing clinical history.

**Fig. 3:**
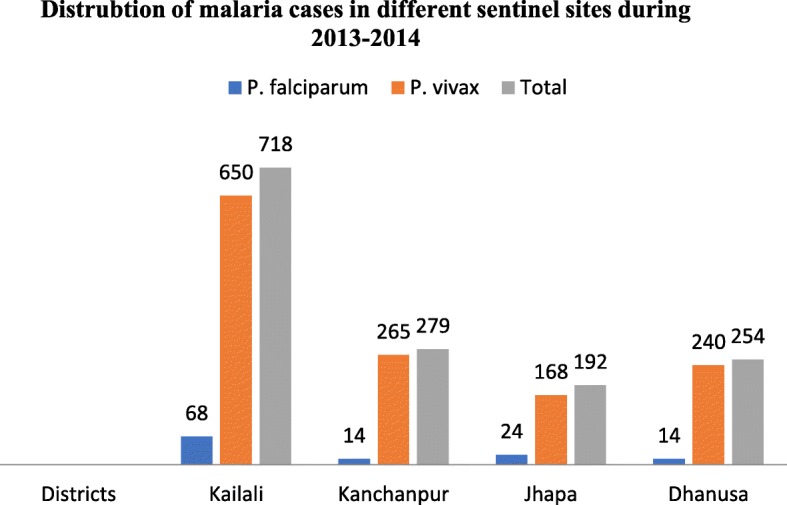
Distribution of malaria cases in different sentinel sites during study period 2013–2014

Twenty-eight (26.4%; 28/106) of them were enrolled into the study with first 3 days’ admission at hospital and 28 days of parasitological monitoring. Among them, total 19 (67.8%; 19/28) enrolled patients were from *Kailali* sentinel site and 8 (28.5%; 8/28) were enrolled from *Kanchanpur* sentinel site (Fig. 3). Four (14.3%; 4/28) cases were excluded from the study as they either could not adhere with the study protocol or failed to follow up.

A total of 24 cases (85.5%; 24/28) completed the study protocol. Of total 24 enrolled cases, 23 (95.83%; 23/24) cases responded well with Artemisinin combination therapy and were classified as ACPR whereas one (4.17%; 1/24) case was microscopically found to have parasitemia at day 28 and therefore classified as LTF (Table 1). However, Kelch 13 was not detected by PCR analysis in all dried blood samples of this particular case. None of the patients who were followed up for 28 days developed any adverse events. Of the 24, five patients were afebrile in day 3 and 19 became afebrile on day 2. Twenty one out of 24 cleared parasites in their blood on day 3 and three cleared parasites on day 7 and one remained parasitemic until 14 days.

**Table 1:** Outcomes of Therapeutic efficacy studies (TES) of artemether-lumefantrine in *P. falciparum* cases

Districts/study year	*Kailali*		*Kanchanpur*		*Jhapa*		Total
	2013	2014	2013	2014	2013	2014	
TES outcomes							
Total *P. falciparum* Screened	46	22	9	5	13	11	106
TES enrollment cases	13	6	7	1	1	0	28
Lost to follow up/Drop out	3	0	0	0	1	0	4
TES completed	10	6	7	1	0	0	24
Adequate Clinical and Parasitological Response (ACPR)	9	6	7	1			23
Early Treatment Failure (ETF)	0	0	0	0			0
Late Treatment Failure (LTF)	1	0	0	0			1

Note: None of the patients who were followed up for 28 days developed any adverse events. Of the 24, five patients were afebrile in day 3 and 19 became afebrile on day 2. Out of 24 patients, 21 had cleared parasitemia on day 3 and three cleared parasitemia on day 7 and one remained parasitemic until 14 days

PCR analyses of filter paper blood samples for K13 mutation at Mahidol University, Bangkok, Thailand showed all the parasites were of wild type and had no detectable mutation on K13 region.

## Discussion

This is a first study in Nepal assessing the therapeutic efficacy of ACTs to inform the national malaria treatment guidelines. In this study, only one case with late parasitological failure was found, however, it did not show any resistance markers for *P. falciparum*. Microscopically, a late treatment failure was detected in one case out of 28. In this case, the clearance of parasitemia was found on day 28. Among 24 *P. falciparum* infected patients who completed the study, majority (83.3%; 20/24) showed no parasitemia in day 3 following administration of artemether-lumefantrine. Low level of treatment failures was consistent with the studies in Ethiopia [27] and India [28]. Contrastingly, however, higher treatment failure (6.4%) was reported in Laos [29].

Nepal has achieved a significant progress in malaria control and treatment in recent years [30, 31], unlike neighboring countries such as Bangladesh which reported a clustering of malaria cases in hilly regions, with emergence of chloroquine and sulfadoxine-pyrimethamine resistance [32, 33]. Following the reports of resistance, artemether-lumefantrine was used as a first line drug for the treatment of uncomplicated falciparum malaria in Bangladesh in 2007 [32]. In 2005, sulfadoxine-pyrimethamine resistance was detected in border areas [34] of Nepal that led to a revised National Malaria Treatment protocol which entailed adoption of Artemisinin combination therapy (artemether and lumefantrine) as a first line drug for the treatment of uncomplicated falciparum malaria [6, 7].

Artemisinin combination generally has a high cure rate, typically exceeding 95% [35]. Our findings showed that the asexual forms of *P. falciparum* are readily cleared after the administration of artemether-lumefantrine (Coartem®) and was consistent with the standard cure rate. These results are consistent with the studies carried out in India [36], and various regions of Africa [37–40].

In PCR analysis, no mutation was detected in Kelch 13 propeller region. K13 propeller region is associated with resistance to Artemisinin [23]. In this study, only one case was found to be resistant to ACTs identified by microscopy; however, it did not show any Artemisinin resistance marker by the PCR.

## Strengths and limitations

This study was jointly conducted by WHO and Epidemiology and Disease Control Division (EDCD) of Nepal to determine the therapeutic efficacy of ACTs in malaria endemic districts of Nepal. Nevertheless, a low prevalence of malaria and *Plasmodium falciparum* to test therapeutic efficacy compromised the statistical power. While the emergence and potential spread of Artemisinin resistance from the GMS can become a public health disaster, findings from this study can reassure the current treatment guidelines with ACTs in Nepal. Future studies to update on the resistance markers can become valuable to assess the efficacy of ACTs.

## Conclusion

A high percentage of cases (95.83%; 27/28) with uncomplicated falciparum malaria, enrolled in this study showed adequate clinical and parasitological response, with only one case of parasitological failure. This study also showed that Artemisinin combination therapy (artemether and lumefantrine in combination) at the dose prescribed by National malaria treatment protocol was still effective. However, further continued monitoring is required in the context of emerging Artemisinin combination therapy resistance in the Greater Mekong Sub-region. Based on this study, Artemisinin combination therapy (ACT) is still effective against *Plasmodium falciparum* in Nepal and should be continued as a first line drug against *P. falciparum.* Nevertheless, therapeutic efficacy using both microscopy and PCR in future are necessary to monitor K13 mutation, a hallmark of Artemisinin resistance in *P. falciparum*.
